# Editorial July 2021

**DOI:** 10.1093/braincomms/fcab150

**Published:** 2021-07-24

Welcome to Volume 3, issue 3 of *Brain Communications*. While we are still struggling with the pandemic here in the UK and worldwide, things are looking somewhat brighter with vaccinations allowing more interactions with loved ones and eventually maybe even in person scientific conferences on the horizon. I almost miss the bad coffee and jet lag that comes with the chance to chat with colleagues. But there are a few silver linings for the field from this horrible experience including the wider reach of online meetings. The organizers of the recent Brain Conference kindly sent us a Field Potential article describing their experience of hosting this conference which allowed us to reach people worldwide with the online format.^1^ We have also continued to receive excellent translational neuroscience papers at the journal and have started to see clusters of papers on popular and important topics in the field. We recently highlighted a group of papers on biomarkers (https://academic.oup.com/braincomms/pages/biomarkers-collection) with an accompanying editorial by Associate Editor Dr Alberto Lleó.^2^

One of the many downsides of the pandemic for our field is that is has brought into even starker relief some of the systemic problems we face as scientists. Early career researchers (ECRs) have been hit particularly hard by the lab shutdowns and the economic downturn and resultant decrease in some of the funding streams. These difficulties for ECRs are not new, however, and one of the things I think we do poorly in academic neuroscience is help people transition out of academia at different career stages. There are not enough positions at the ‘top’ of academic neuroscience for all of our PhD students and postdoctoral researchers, but the skills gained in scientific training are of great value beyond academia in many areas including science policy, biotech, teaching and of course publishing. In the past few months, several ECRs have approached our team at *Brain Communications* asking for experience in the scientific publishing field. One of our goals is to promote career development of neuroscientists, so in response, we have decided to start a *Brain Communications* Observers scheme. In this scheme, led by our Scientific Editor, Dr Manuela Marescotti, we will demonstrate some of the day-to-day tasks of an editor and discuss the journal’s wider strategy with observers. With this programme, we aim to help observers understand the process of publishing a paper, give them insight into working as part of the editorial team, and enhance transparency of our editorial processes. At our last Editorial Board meeting, there were also fantastic ideas including a ‘reviewer academy’ to support ECRs in learning to review papers and giving them credit for doing so. Keep an eye on our website for more details.

For those of you wondering when we will have our first impact factor, the short answer is I don’t know and, to a certain extent, I don’t care. One of our founding principles is that we promote publication of rigorous neuroscience studies without requiring that every study be completely novel. We welcome replication studies, well-substantiated negative results, and repeating key experiments in different model systems. These studies by their nature may not be cited as highly as completely new findings, but we firmly believe it is important that they be published to help move the field forward and enhance credibility in neuroscience. Thus, we are NOT trying to drive a high impact factor by restricting acceptance based on novelty. I appreciate that for career progression, publishing in ‘high impact’ journals is important for most of us despite our institutions and funders signing up to initiatives like the San Francisco Declaration of Research Assessment (DORA https://sfdora.org/). Luckily for us, our fantastic authors have sent solid and super-interesting work that is already being well cited, so we are predicted to have a respectable impact factor, when it is assigned, despite not striving for this as a goal.

The cover image for this issue is from Massey et al.^3^ showing a lovely mouse hemibrain section with nuclei stained blue and three different RNA labels in their study of modifiers of risk of sudden unexpected death in epilepsy.

Thank you for your continued contributions to the journal!

Tara Spires-Jones, Edinburgh, UK
